# Life Origination Hydrate Hypothesis (LOH-Hypothesis)

**DOI:** 10.3390/life2010135

**Published:** 2012-01-04

**Authors:** Victor Ostrovskii, Elena Kadyshevich

**Affiliations:** 1 Karpov Institute of Physical Chemistry, Vorontsovo Pole str. 10, Moscow 105064, Russia; 2 Obukhov Institute of Atmospheric Physics RAS, Pyzhevsky str. 3, Moscow 119017, Russia; E-Mail: kadyshevich@mail.ru

**Keywords:** LOH-hypothesis, life origination hydrate hypothesis, living matter origination thermodynamic grounds, life origination chronology, nucleotide formation thermodynamics, life origination chemism and mechanism

## Abstract

The paper develops the Life Origination Hydrate Hypothesis (LOH-hypothesis), according to which living-matter simplest elements (LMSEs, which are N-bases, riboses, nucleosides, nucleotides), DNA- and RNA-like molecules, amino-acids, and proto-cells repeatedly originated on the basis of thermodynamically controlled, natural, and inevitable processes governed by universal physical and chemical laws from CH_4_, niters, and phosphates under the Earth's surface or seabed within the crystal cavities of the honeycomb methane-hydrate structure at low temperatures; the chemical processes passed slowly through all successive chemical steps in the direction that is determined by a gradual decrease in the Gibbs free energy of reacting systems. The hypothesis formulation method is based on the thermodynamic directedness of natural movement and consists ofan attempt to mentally backtrack on the progression of nature and thus reveal principal milestones alongits route. The changes in Gibbs free energy are estimated for different steps of the living-matter origination process; special attention is paid to the processes of proto-cell formation. Just the occurrence of the gas-hydrate periodic honeycomb matrix filled with LMSEs almost completely in its final state accounts for size limitation in the DNA functional groups and the nonrandom location of N-bases in the DNA chains. The slowness of the low-temperature chemical transformations and their “thermodynamic front” guide the gross process of living matter origination and its successive steps. It is shown that the hypothesis is thermodynamically justified and testable and that many observed natural phenomena count in its favor.

## 1. Introduction

The problem of the border between the animate and inanimate has been discussed since 1935 when Stanley pioneered isolation and crystallization of the tobacco mosaic virus. Different opinions expressed in this discussion are considered in [1]. Apparently, once nucleic acids originated and propagated and a medium appropriate for their existence and replication had appeared, the appearance of cellular life was merely a matter of time; if a living system were devoid of nucleic acids, with its protein content preserved, the vital activity would surely cease; if a living system were devoid of its protein, with the nucleic acids preserved, the vital activity of the system supplied with nutrients might normalize with time.

Thus, the occurrence and reproduction of nucleic acids (desoxy-ribonucleic acid (DNA) and ribonucleic acid (RNA)) is the principal feature of living matter. Their molecules represent alternating phosphate–sugar chains, in which a hydroxyl of each sugar group is substituted with a so-called nitrogen base (N-base). As sugars, desoxy-D-riboses (DDR) and D-riboses (DR) enter the molecules of DNA and RNA, respectively. As N-bases, pyrimidines (cytosine (Cy), thymine (Th), and uracil (U)) and purines (guanine (G) and adenine (Ad) and, more rarely, xanthine (X), hypoxanthine (Hx)) and some quite rare N-bases are known. DNA molecules contain no U, and RNA molecules contain no Th. Note that N-bases can exist in deprotonated or protonated form depending on the medium acidity. The product of interaction of a ribose with an N-base and with a phosphate group is termed nucleotide, and the product of interaction of an N-base with a ribose is termed nucleoside. Below, N-bases, riboses, nucleotides, and nucleosides are termed living matter simplest elements (LMSEs) and DNA- and RNA-like molecules and proto-cells are termed simplest living matter.

In living organisms, DNA molecules are, at least most of the time, in the state of dimers, termed double helixes. The spatial arrangement of different components in the molecules of nucleic acids is strictly defined. For each organism, the occurrence of DNA molecules of a definite composition is the characteristic feature. It is commonly accepted that the heritage of living organisms is determined by the sequence of the N-bases in the DNA molecules.

The history of the scientific interest to the living matter origination process began in 1924, when A. Oparin announced the first-ever naturalistic hypothesis of life origination [2,3]. This hypothesis became the starting point for numerous works dedicated to this intriguing problem. Oparin thought that living matter originated at ground–atmosphere or water–atmosphere phase boundaries as a result of the following evolution sequence:

Simplest organic molecules → protein precursors → protein →

→ organized protein bodies (coacervates) → metabolizing living matter→ unicellular organisms (1)

It is seen that Oparin placed emphasis on proteins as the precursors of living matter. It should be remembered that, in 1924, when Oparin began to develop his hypothesis, nucleic acids were not known and that, in the early 1950s, when Oparin presented the life origination hypothesis in the book, the fundamental importance of the nucleic acids for the phenomenon of life was not absorbed by the scientific community. Oparin thought that the living matter entropy is so small that no decrease in the enthalpy could make the free energy change negative in chemical transformations of mineral substances to living matter. Therefore, he believed that external energy in the form of electric discharges, heat of underground thermal springs, *etc.* is necessary for such transformations.

Meanwhile, the almost 60-year experiments started by S. Miller and H. Urey [4,5] and continued by their numerous followings showed that, in gaseous mixtures of CH_4_, NH_3_, H_2_, and H_2_O and of other compositions and under action of electric discharges, different organic substances can be produced and that their hydrolysis can lead to formation of biologically important substances; however, none of the many hundreds of experiments allowed synthesizing either nucleotides or nucleosides.

In the 1980s, physicist and biologist L. Blumenfeld calculated [6,7,8] the living matter entropy on the basis of the simple generalized approach of statistical physics and came to the unambiguous conclusion that “…according to physical criteria, any biological system is ordered no more than a rock piece of the same weight” ([7], p. 90). Somewhat later, the standard values of the enthalpy of formation (Δ_r_H^0^) and of the entropy (S^0^) were obtained experimentally for different biologically active substances [9,10,11,12]. Some of them are given in [1], where we calculated the standard (Δ_i_G^0^) values for a number of reactions leading to formation of N-bases and riboses from minerals. It was stated that no external energy is necessary to synthesize biologically active substances from some minerals, because the free-energy changes in such reactions are negative and rather great in magnitude and, thus, the principal theoretical ground for the Oparin’s hypothesis was broken.

The life origination hypotheses developed by Oparin and his followers was also criticized from another standpoint by R. Shapiro [13]. He believes that the production of an information-bearing homopolymer within a complex mixture by chance cannot be excluded, but if such an event was required to start life, then its origin would have been an extremely improbable accident, and prospects for life elsewhere would be diminished. According to his opinion, “…a more likely alternative for the origin of life is one in which a collection of small organic molecules multiply their numbers through catalyzed reaction cycles, driven by a flow of available free energy”[14]. Similar views on life origination problem are available in the literature (e.g., [15,16,17]).

Meanwhile, DNA and RNA molecules are very long and include chemical elements chosen very selectively from the environment, the N-bases, riboses, and phosphate groups are located in a strictly determined sequence, the N-bases are limited in sizes in spite of the occurrence of chemically active groups in their compositions, and have some other special features. Therefore, such molecules could originate only under very specific conditions. These peculiarities of nucleic acids led us to the idea that they originated inside such a natural honeycomb matrix which selectively absorbed source substances of definite composition and the cavities of which limited the chemical accretion of each individual LMSE, but did not hamper their polycondensation.The strict directedness of this process resulted from the slowness of the chemical reactions, *i.e.*, from such low temperatures that they proceeded one-by-one in the direction of decreasing in the free energy of the system until the structural cavities are completely filled.

Such a mechanism could be realized only in the absence of heat flows, electrical discharges, and weather perturbations capable of destroying any order rather than of creating and maintaining it. Therefore, formation of DNA and RNA molecules from simple mineral substances by Oparin’s mechanism at water–atmosphere or ground–atmosphere boundary represents an extremely low-probability event, which cannot be realized in practice. In addition, Oparin’s hypothesis gave no realistic assumption on a possible cause of the phenomenon of monochirality of biologically active substances.

We developed the OK-hypothesis of the Universe development for the period from the explosion of the presolar star to origination of cellular living matter (OK is the abbreviation of the family names of the authors). This hypothesis includes the Solar System formation hypothesis (PFO–CFO hypothesis) [18,19,20,21,22,23,24,25], hypothesis of formation of natural gas and some other localizations of minerals [20,25,26], and the Life Origination Hydrate hypothesis (the LOH-hypothesis) [1,27,28,29,30,31,32,33,34]. Several decades ago, the problem of living matter origination was divided into two sub-problems: where did living matter originate, at the Earth or anywhere in the Universe beyond our planet, and how, *i.e.*, by what mechanism, did it originate? We leave the first sub-problem out of our consideration and discuss the living matter origination mechanism, which can be applied, in our opinion, to any Universe region, where the appropriate conditions exist.

When developing the OK-hypothesis, we adhere to the following common principles: (1) The gross-scale processes in nature proceed progressively in the direction of decrease in the free energy in the Universe subsystems that can be approximated as the isolated ones. (2) All natural phenomena proceed as a result of regular and inevitable transformations regulated by the universal physical and chemical laws. (3) The Newton principle of simplicity (“…for Nature is pleased with simplicity, and affects not the pomp of superfluous causes”). (4) The principle of repetition of supposed events and of the presence of individual features in the reproduced events (Nature created many similar but somewhat differing events and no unique event without close analogues.). (5) The principle of the unity of the event point. Separation of an event into several sub-events proceeding in different points with subsequent interaction between the sub-events decreases the probability of the resulted event, because it decreases many-fold the degree of repetition of the event as a whole. (6) Nature makes no jumps (*Nature non facit saltus*, in Latin).

Just as a result of the directedness of natural phenomena, researchers are principally capable of mentally doubling back on the course of Nature and, thus, revealing the main milestones in Nature’s progression. A naturalist must search for a “hook” in the environment in order to catch onto it and, having the thermodynamic laws as a guiding thread, to guess the logics used by Nature in its development.

The LOH-hypothesis is based on the application of the well-known natural phenomenon of formation–destruction of mineral honeycomb guest–host structures, namely, of the so-called gas-hydrate structures, to solving the living-matter origination puzzle. The combination of the below-considered size agreement between the hydrate and DNA structures and specific features of this phenomenon was the “hook” that initiated our thinking over this puzzle. Just the experimental results [35,36,37] on the water interaction with functional polymers led us to the idea of the formation of hydrate-like structures around functional groups of polymers in the water environment. The same phenomenon was used by us as the basis for the hypothetic explanation of physical and chemical mechanisms of some principal intracellular processes, including mitosis of eukaryotes and binary fission of prokaryotes [38,39,40].

The place of the LOH-hypothesis among other hypotheses of living matter origination was detailed in [1] and other earlier publications by these authors. Therefore, we limit ourselves now by referring to more recent available reviews [41,42] dedicated to the life origination problem, by several above-given remarks relative to the well-known Oparin’s hypothesis that has a number of followers to the present day, and by the underwritten digest of some earlier works relevant to the problem of living matter origination.

In the context of Oparin’s hypothesis, such sources of external energy for syntheses of biologically important substances on the young Earth’s surface as impact shocks or ultraviolet light and extraterrestrial objects, namely, comets, meteorites and so on, have been discussed by Miller and Orgel [43], Schopf and Walter [44], Walter [45], Chyba and Sagan [46], and others. An original idea was proposed by Gold [47]. According to his opinion, life first originated not on the Earth’s surface but many kilometers under it and the most ancient living matter had been independent of the surface circumstances and solar energy. According to Gold, the intra-terrestrial life had been supplied with energy from chemical sources due to fluids migrating upward from deeper levels in the Earth. We see that Gold was affected by Oparin’s hypothesis and also believed that exterior energy is necessary for living matter origination from minerals. Gold's ideas were supported by Holland [48] and D’Hondt *et al.* [49], who showed that, deep under the Earth's surface, there are deposits that cannot be formed without bacterial activity; meanwhile, living matter as such was not identified in these works.

Since the papers by Nagy *et al.* [50] and Fitch and Anders [51], who had found organic particles embedded in minerals in the so-called Orgueil meteorite, the hypothesis of extraterrestrial origin of the Earth's living matter (the so-called Panspermia) has received a rather wide acceptance. Generalizations of the results obtained in these works were performed by Bernstein *et al.* [52], Rhawn [53], and Wainwright [54]. This hypothesis continues to have its followers . However, it belongs to the problem “where did living matter originate?”, rather than to the problem “by what mechanism did living matter originate?” Therefore, we do not consider this hypothesis.

In the 1980s, it was discovered that RNA shows catalytic properties in biological systems [55,56]. These works had led to the hypothesis that living matter on the basis of RNA, the so-called RNA World [56], preceded the present-day living matter. Somewhat later, the model of auto-catalytic replication of RNA was proposed [57]. The notions regarding the RNA World were supported in works [58,59,60,61,62]. During the last 10–15 years, it was repeatedly shown, e.g., by Orgel [62,63] and some other authors (e.g., by Li and Nikolaou [64]), that enzymes are not necessary for replication of different organic molecules and that not only nucleic acids but also simpler organic substances are capable of self-replicating. According to [65], RNA molecules in the RNA World had simultaneously functioned as the genetic material and enzyme-like catalysts.

Below, we briefly consider an important and rather intriguing question on dating the historic period when the first simplest living organisms appeared on Earth. This question was recently [66,67] developed as comprehensively as the current knowledge of the Archaean history of the Earth allows. According to [68,69], the early divergence among prokaryotes was 3970 Myr ago (molecular time) or 3800 Myr ago (fossil time) and the origin of eukaryotes was 2730 Myr ago (molecular time) or 2150 Myr ago (fossil time). Note that the dates of divergence among prokaryotes were recently called into question. The matter is that this conclusion was made on the basis of indirect data on the isotopic composition of the carbon inclusions within the apatite crystals mined from the Greenland Archaean beds. Meanwhile, according to [70,71], the apatite crystals and carbon inclusions could be formed much later as a result of metamorphism influenced by the hot fluids. We see that the molecular time and the fossil time differ rather significantly and that the periods of the starts of the Earth prokaryotization and eukaryotization are known rather approximately. These conclusions are not solely relative to the prokaryotes and eukaryotes ages. Fossil time measurements led to the conclusion that the earliest localities of invertebrate animals lived 600–550 Myr ago and that most of the species of invertebrate animals occurred in Cambrian period, *i.e.*, their age is less than 510 Myr. However, measurements of the molecular time give a period of 1500–1200 Myr ago for the development of the first Metazoa colonies and lead to the conclusion that the main branches of the invertebrates occurred many hundreds of millions of years before Cambrian [66]. Note that, according to [72], multicellular organisms appeared independently in the Earth history no less than 24 times.

The data given in this section show that today’s knowledge on the positions of different events on the time-scale are rather approximate. Nevertheless, it is known that, according to the general trend, the later along the time scale a species appeared on Earth, the more complicated is its internal cellular constitution and the more complicated is its genome [66].

## 2. The LOH-Hypothesis Content

### 2.1. General Principles

The LOH-hypothesis was published, at different steps of its development, in [1,26,27,28,29,30,31,32,33,34,40], as was mentioned above, and was presented to a number of audiences around the world [73,74,75,76]. This paper is mainly dedicated to the thermodynamic grounds of the LOH-hypothesis and to discussion of the naturalistic essence of the LOH-hypothesis and of its relation to Darwin’s hypothesis. The discussion is performed in the context of the principal requirements underlying the sub-hypotheses entering the OK-hypothesis and in the context of the available data on the life origination chronology.

An analysis of the phenomenon of living matter led us to the conclusion that the hypothesis of its origination should take into account the following statements: (1) The DNA occurrence and reproduction is the principal feature of living matter; the proteins are side products. (2) The Earth’s living matter had originated on our planet from inorganic and the simplest organic minerals as an inevitable product of the atomistic world. (3) Stable undisturbed conditions favoured living matter origination. (4) The reacting system transformed so slowly that it passed all possible states step by step in the direction of gradual decrease in the Gibbs free energy. (5) The diversity of the available forms of living matter is caused mainly by some variations in the parameters of the native medium.

The key event of life origination is DNA and RNA formation from strictly definite mineral substances, which are termed below (up to the subsequent concretization) by the collective term “nutrition”. Various proteins are the side products of DNA and RNA formation. DNAs and RNAs form in common, and some amino-acids form together with them from the same source substances. Any localization that is appropriate for formation of DNA and RNA molecules is capable of producing a multitude of DNA- and RNA-like molecules with different sequences of N-bases. Living matter originated repeatedly in the course of Earth’s history. The cells originated within the localizations (incubators) where DNAs and RNAs formed. The longer such an incubator existed, the lengthier were the DNA and RNA molecules and the more complicated cells formed within them. The cells could begin to live independently on leaving the incubator or after breaking of the latter. Therefore, as a rule, the more complicated the cellular structure and DNA of any organism, the longer the history of this DNA and genealogy of this organism.

### 2.2. From Minerals to DNA- and RNA-Like Molecules

It is well-known that alkanes are capable of reacting with dilute HNO_3_ with formation of nitro-alkanes as the primary product. This reaction was discovered by Konovalov [77] in 1888 and bears his name. One would expect that this reaction could proceed within the methane-hydrate phase. Under room temperature, Konovalov’s reaction proceeds extremely slowly, and its rate can be measured only above 400–500 K depending on the hydrocarbon nature. The nitration rate for methane is the lowest one as compared with that for other hydrocarbons. Nitro-methane is capable of subsequent reacting with methane and NO_3_^−^-ions giving more complicated products. Of course, these reactions proceed very slowly on the scale of usual laboratory experiments, but the slowness of these natural reactions and the constancy of the conditions are necessary for the thermodynamic controlling of the sequence of the reaction steps, for uniform packing of all structural cavities by guest molecules of any one composition, and for the gradation in the transformations of the guest molecules in the direction “from the simple to the complex” over the entire volume of a gas-hydrate crystal. Nature has nowhere to hurry. Also, for any planet, 500 Myr after its formation, the underground regions deepened by several kilometers have the most stable conditions.

The essence of the LOH-hypothesis is as follows (see also [1,31,32]). The LMSEs, DNA- and RNA-like molecules, and proto-cells originated (and, may be, originate in our days) from CH_4_ (or another CH_4_-hydrocarbon), niter, and phosphate under the Earth’s surface or seabed within honeycomb hydrate structures. First, N-bases, riboses, and nucleosides formed from CH_4_ and nitrate-ions as a result of NO_3_^−^ diffusion into the hydrate structure, and then nucleotides and DNA- and RNA-like molecules formed as a result of phosphate-ion diffusion into the structure. N-bases and phosphate groups were localized within the large and small structural cavities, respectively. NO_3_^−^ ions diffused into the gas-hydrate structure, because CH_4_ molecules reacted selectively with them, and thus these reactions revealed themselves similarly to a pump. Riboses can be housed within small or large cavities. (The large cavities of hydrate structure H are somewhat “more roomy” than those of structure II, and we cannot exclude that structure H is the matrix for LMSE formation.) The reactions of LMSE formation proceeded at significant depths under a CH_4_ pressure of several MPa and temperatures of 273 ± 20 K, *i.e.*, under the conditions when CH_4_-hydrate is stable.

**Figure 1:**
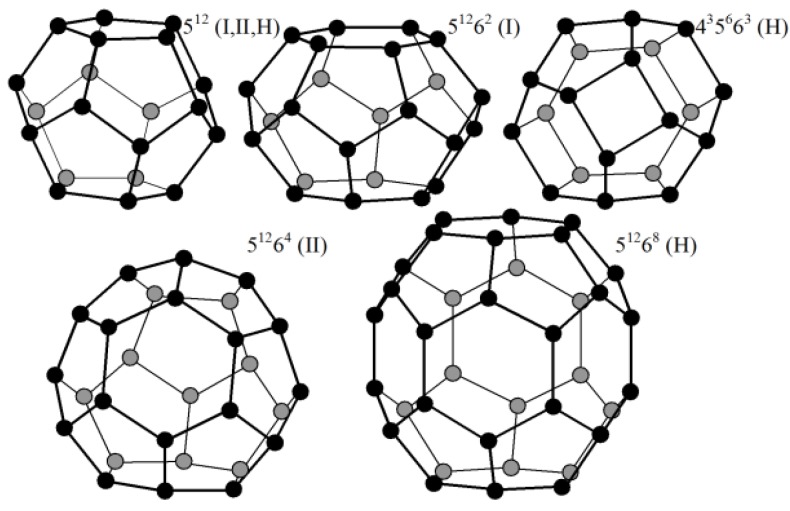
Hydrate cavities of structures I, II, and H.

Figure 1 is designed in scale; it presents the gas-hydrate cavities of hydrate structures I, II, and H. Structures I, II, and H contain 5^12^ and 5^12^6^2^, 5^12^ and 6^4^, and 5^12^, 4^3^5^6^6^3^, and 5^12^6^8^ cavities, respectively (the lower-case figure means the number of the edges of a facet and the superior figure means the number of such facets that terminate the corresponding cavity) [78]. Each of structures I and II contains cavities of two types, and H structure contains cavities of three types. In Figure 1, each vertex responds to the O atom of a water molecule and each edge responds to the sum of O–H valence bond of any H_2_O molecule and H····O hydrogen bond of this H_2_O molecule with any adjacent H_2_O molecule. Methane hydrate structures can exist only under the condition that some guest molecules are housed within no less than 80% cavities of, at least, any one type; otherwise, the loose structure collapses and transforms to the usual thick ice structure. The hydrate structures are capable of reconstructing from one to another. The structure type depends on the size of the guest molecule or on the sizes of the guest molecules, if molecules of two or three types are located within the cavities, each guest type within the cavities of any one size. According to our two-dimensional consideration, structure II is appropriate to house DNA and RNA [1,31,32]. A three-dimensional modeling performed by Alexander Dzyabchenko confirmed this conclusion. This work is in the process of development, and its details will be published later.

In [79,80], it was demonstrated that, over the globe, a great number of underground and under-seabed methane-hydrate localizations exist and, on frequent occasions, localizations of niters are in their vicinities [1,32]; nitrate-ions are also dissolved in different water basins, including underground ones. Localizations of phosphates exist all around the world and, if there are doubts in the mineral origin of some of them, it is commonly accepted that apatite Ca_5_Y(PO_4_)_3_ (Y = F, Cl, or OH) is of mineral origin [81]. In addition, phosphate-ions are dissolved in different water sources and it is not impossible that the young Earth had contained elementary phosphorus, which had slowly reacted as follows (the heat effect is given for the standard conditions); at least, there are no doubts that this reaction is thermodynamically feasible [81]:

P_4_ + 16 H_2_O = 4 H_3_PO_4_ + 10 H_2_ + 1306 kJ (2)

**Figure 2:**
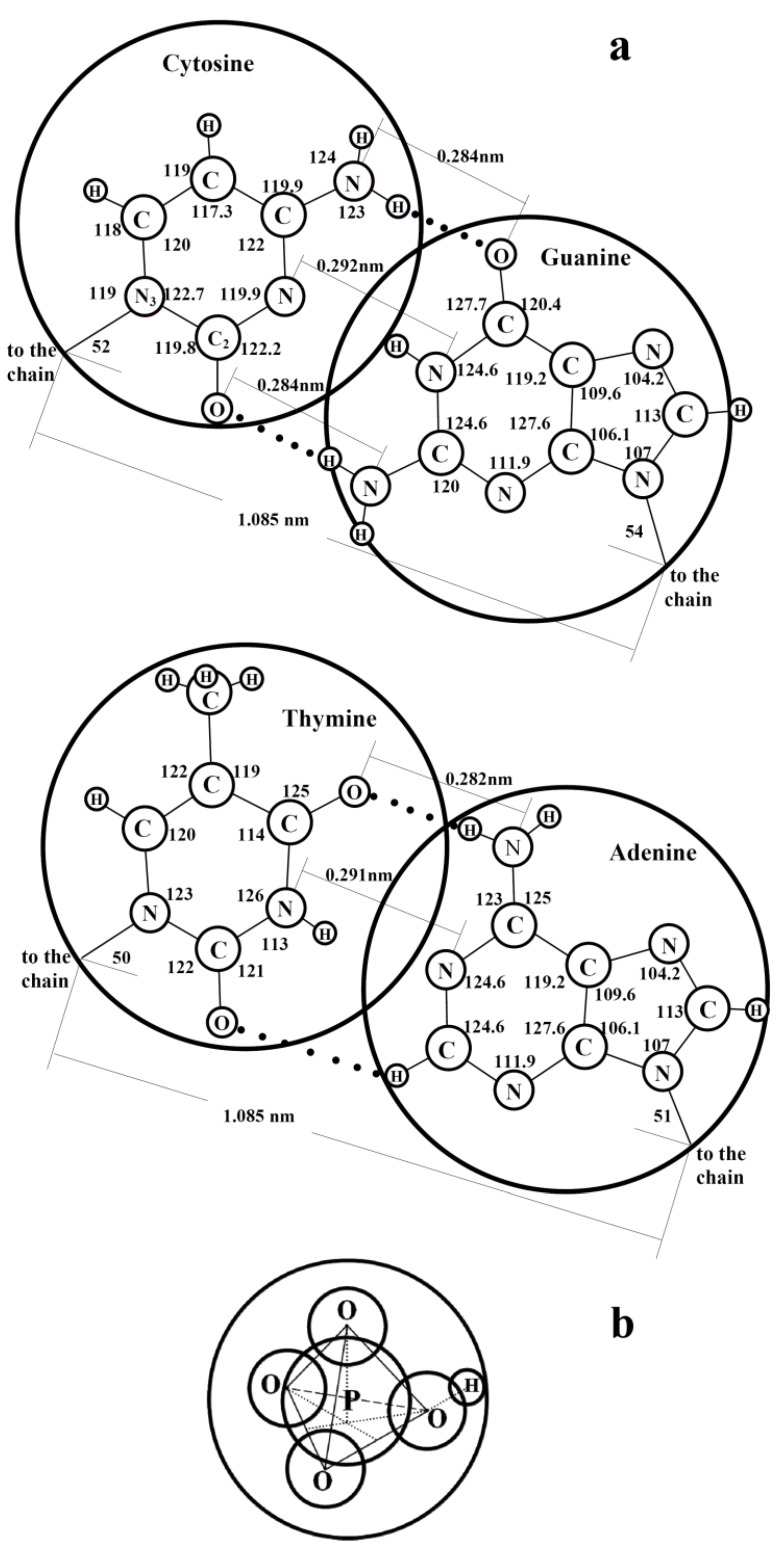
(**a**) Scaled schematic representation of pairing between N-bases of two DNA molecules in the double helix structure; the valence angles are given in degrees, circles of diameter 0.69 nm correspond to the free diameter of the large cavity in hydrate structure II. (**b**) Scaled schematic representation of a phosphate group inside a small cavity of hydrate structure II; a circle of diameter 0.48 nm corresponds to the free diameter of the small cavity.

Figure 2 is designed in scale; the diameters of the large and small circles respond to the free volumes of the hydrate structure II large and small cavities, respectively. It is seen that the sizes of N-bases and phosphate-ions correlate well with the sizes of the large and small cavities, respectively. Thus, in this hydrate structure, the large cavities are as if created as the moulds for the N-bases and the small cavities are as if created as the moulds for the phosphate-ions. In order that the hydrogen bond would arise between purine and pyrimidine bases, the distance between them should be strictly fixed. Apparently, just the hydrate structure with its systematically-located large cavities creates the geometric conditions necessary for such binding. Our two-dimensional consideration gives grounds to assume that DNA molecules originate within the hydrate structure by pairs and form the double helixes at the step of their origination from mineral substances. If the LOH-hypothesis is correct and the LMSEs had originated actually within hydrate structure II, the composition of N-bases within the large cavities at their full filling cannot be identical. The matter is that full filling of any one cavity can hamper full filling of a neighbor one because atoms of any two adjacent molecules either interact with each other and are at a distance of the valence bond or do not interact with each other and are at a longer distance, which is no less than the sum of their Van der Waals radii. Thus, the guest atoms housed within a cavity can hamper full filling of the space of the neighbor cavities by guest atoms if a valence bond between the guest atoms of these neighbor cavities does not form. If two molecules of purine bases were formed inside two neighboring large cavities of the hydrate structure, the distances between the oppositely polarized groups belonging to these two molecules would be smaller than the equilibrium ones and, therefore, the polar groups of these molecules would inevitably “turn away” from each other.

These features stimulate nonrandom locations of N-bases and provide nonrandom specific sequences of the N-bases in the DNA and RNA-like molecules, which originate as a result of polycondensation of nucleotides formed from riboses, phosphate-ions, and N-bases.

Due to one more cause, the common structure of the methane-hydrate deposits provides a nonrandom arrangement of atomic groups in the DNA- and RNA-like molecules. The methane-hydrate deposits do not represent compact homogeneous strata similar to the strata of coal or salt. They are associated with underground sandy or frothed and then solidified rocks and are similar to a hoar-frost, which locks the sand and transforms it to a rather dense monolith mass or fills up the porous rock structure. The methane-hydrate crystallites have millimeter-range sizes and are similar to three-dimensional snowflakes or fine ice hail impregnated with methane and integrated with the parent mineral material. Apparently, each such a methane-hydrate crystal can be a source of one DNA-like or RNA-like molecule; the processes that proceed within any one crystal are independent of the processes that proceed within any other crystal. The following features are important: First, in each crystal, the process is slow, thermodynamically controlled, and capable of going up to full filling of each large cavity. Second, within large cavities, uninuclear pyrimidines and two-nuclear purines with different side groups are housed and any two atoms not bound chemically to each other are arranged at a distance no less than the sum of their Van der Waals radii. Therefore, when the large cavities are already almost filled with purine and pyrimidine nuclei and some side groups are already produced, a specific situation should arise. Namely, the appearance of any additional side group (term it “key” side group) in the composition of one of the N-bases determines unambiguously the chemical nature and geometrical positions of the side groups that should finish filling the hydrate structure over a significant portion of the crystal or even over its entire volume. The unambiguity of the composition of these finishing side groups follows from the chemical possibilities (*i.e.*, from the nature of the source chemical substances) and geometric factors inherent in the system under consideration. One more feature should be noted. In each methane-hydrate crystal, the “key” side group may be produced at a random crystal point and, apparently, may be of different chemical composition. Therefore, methane-hydrate crystals of any deposit are capable of producing a multitude of different nucleic acids.

Thus, the content of the three previous paragraphs shows, in our opinion, that a combination of the matrix effect with the effect of thermodynamic controlling of the chemical reactions in the system under consideration can create conditions for specific, and by no means random, locations of N-bases in the resulted DNA-like and RNA-like molecules and that a variety of different and specific nucleic acids can, apparently, originate in any one methane-hydrate localization. The importance of nonrandom arrangement of N-bases was stressed in [82,83].

Polycondensation of N-bases, riboses, and phosphate groups is stimulated by crystallization of the emitted waters with the methane-hydrate formation caused by the excessive CH_4_ pressure. From Figure 2, it is seen that phosphate ions can be housed within the small cavities. Apparently, just the hydrate structure provides the regularity of location of N-bases, riboses, and phosphate groups in the DNA (and RNA) molecules and just the methane chemical inertness provides the selectivity of methane interaction only with nitrates and phosphates. Methane-hydrate manifests itself in this natural process as a selective reactive membrane.

The LOH-hypothesis allows explaining the mysterious phenomenon of monochirality of all known species of living matter. The fact is that, the RNA and DNA molecules contain in their composition only D-riboses and desoxy-D-riboses but not L-riboses and desoxy-L-riboses, respectively. Meanwhile, D-forms and L-forms of any substance are identical in their chemical compositions, reaction abilities, and sizes and any chemical reaction of their formation produces them in equal numbers. Such isomeric molecules have only one distinctive feature; namely, they are the mirror images of each other. Many physicists, biologists, and chemists tried unsuccessfully to guess the riddle about the cause of monochirality. Even such nontrivial causes as the fundamental asymmetry of the Universe and the weak interaction causing nuclear beta decay were discussed (e.g., [84,85]).

According to our hypothesis, the DNA and RNA monochirality is a natural inevitable consequence of the gas-hydrate matrix geometry: within hydrate structure, only D-riboses, but not L-riboses can “touch” both an N-base and a phosphate group, by the active groups of their structures. Apparently, only under such conditions, each phosphate group is capable of “touching” two riboses and thus to join together two nucleotides and to ensure lengthening of DNA or RNA chains. At temperatures around 270 K, when methane-hydrate is stable, CH_4_ reacts utterly slowly on the laboratory-experiment time-scale. The subsequent interactions of the intermediate products with nitrate-ions and methane and the diffusion processes are also extremely slow. It can be expected with a high degree of certainty that increasing of the length of DNA-like (RNA-like) molecules is rather long-lasting on the geological time-scale. The longer the process is in any one locality, the lengthier are the nucleic acids. In different localities over the globe, the processes that led to DNA and RNA origination could start in different epochs and, although the laws of reacting were universal, the directions of the reactions could be not identical as a result of differences in the ambient conditions. Two last conclusions are rather important, and we attract the attention of researchers to them.

As was said above, the molecules within the cavities grew with time as a result of interaction between CH_4_ and NO_3_^−^-ions in the direction of purine, pyrimidine, and ribose formation up to filling of the cavities and as a result of nucleotide formation and their joining to the polymer chains. There was plenty of time for purine, pyrimidine, ribose, and desoxy-ribose molecules to form, for the purine molecules to meet the pyrimidine molecules and to form purine–pyrimidine hydrogen bonds, and for D-ribose and desoxy-D-ribose molecules to find purines or pyrimidines and to join them through the chemical bonds with formation of nucleoside dimers, which may transform then to elements of DNA dimers or RNA fragments. Phosphates diffused into the hydrate structure by the empty small cavities and could react in the direction of DNA or RNA formation only under the condition of their diffusion into a cavity located between two adjacent cavities filled with two D-riboses or two desoxy-D-riboses. As was said above, we assume that the hydrate-structure geometry allows location only of D-ribose and desoxy-D-ribose between an N-base and a phosphate-group. This does not mean that L-riboses do not form. They form, but enable formation of amino-acids and other side products. Of course, the positions of desoxy-D-riboses and D-riboses between N-bases and phosphates were not housed right away but after a great number of trials and errors. As was said earlier, the reactions within the gas-hydrate structures proceeded step by step so slowly that their directions were controlled by the thermodynamics and they went in the same direction within all cavities. Formation of the molecular chains of DNA and RNA that were produced in reality corresponded to the minimum of the free energy, and, therefore, the acts of exchange by functional groups led steadily to the achievement of the optimum positions. For so complicated reactions governed by thermodynamics, any search for answers to the questions of detailed mechanisms is pointless. For example, we have no means to ascertain, why any functional group joins to the position to which it joins in reality rather than to another one, to which, in our opinion, it could join. Analogously, we have no means to explain, why nitrogen atoms enter the nitrogen bases but do not enter riboses. Consider one more example of such a kind. A ribofuranose structure has four chiral centers. Each of the N-bases of RNA offers multiple sites for attachment of a substituent, and each ribose offers three hydroxyl groups for phosphate ester formation. In spite of the seeming possibility of formation of different products, Nature chooses the connections resulting in the formation of nucleotides and nucleic acids. Very likely, even succeeding generations will never be able to calculate the details of the thermodynamics for such complicated systems. However, we cannot stint Nature of such abilities. In our opinion, all structural details, in principle, are explainable by the chemical affinity and thermodynamics but our chance of achieving this is unattainable in practice. If the interaction of NO_3_^−^-ions with CH_4_ within a gas-hydrate structure were proceeding infinitely long, and if the ambient conditions for different localizations were identical, these processes would lead to formation of nucleic acids of one type. However, such processes are not infinitely long and the ambient conditions are different for different localizations. Therefore, although the directions of the processes are, in general, the same in all localizations, each localization produces at any time a multitude of different DNA and RNA molecules, and different localizations produce different sets of DNA and RNA molecules even under the condition that the processes within them start simultaneously.

Let us formulate an intermediate resume at this step of our consideration.

Thus, (1) DNA and RNA molecules are produced on Earth (and, maybe, on other planets) from only three widely distributed minerals under ambient conditions, invariable over a long time, and at such low temperatures that the direction of the reactions is determined by the thermodynamics; (2) methane-hydrate, as a chemical reagent, gives methane for construction of future DNA and RNA molecules and provides formation of a multitude of similar but somewhat different DNA- and RNA-like molecules in any one localization; (3) the methane hydrate structure sorbs nitrate and phosphate ions but sorbs no other substances, determines the sizes of the molecules that can be produced inside the structural cavities from methane and nitrate-ions, and rejects L-riboses and desoxy-L-riboses; (4) the thermodynamics determines the direction of the processes inside the structural cavities and thus leads to formation of N-bases and riboses; (5) phosphate-ions, as chemical substances in combination with the thermodynamic laws and gas-hydrate structure features, arrange different LMSEs in regular sequences and link them; and (6) the time, temperature, size of methane hydrate crystallites, and other factors determine the length of the chains and their individual patterns.

### 2.3. From DNA- and RNA-Like Molecules to Proto-Cells

In any methane-hydrate deposit, the processes of formation of the LMSEs and DNA- and RNA-like substances led to a decrease in the volume of the CH_4_ deposit and to isolation of significant amounts of water. For the time being, the decrease in the methane pressure was cancelled by soil lowering, water sorbed methane, additional methane-hydrate amounts formed, and the methane-hydrate deposit remained solid. However, with time, a semi-liquid component appeared and its portion steadily increased in comparison with the portion of the solid methane-hydrate. The semi-liquid component represented a highly concentrated prebiotic soup that was an aqueous solution of different organic and organo-phosphate substances. We term this soup super-cytoplasm, by analogy with the cellular cytoplasm. Liquation of the system and formation of the super-cytoplasm could be stimulated by ambient factors, such as diffusion of excessive water and phosphoric acid into the system, a temperature increase associated with global and regional geophysical and geochemical processes or with faint-young-Sun period termination, *etc*. This hypothetic super-cytoplasm contained the DNA- and RNA-like molecules, N-bases, riboses, nucleosides and nucleotides, amino-acids, and other products of methane oxidation by niter. It could hold the hydrate structure, because the gas-hydrate structure is, seemingly, inherent in very concentrated water–organic systems at about 270 K under undisturbed conditions even at the atmospheric pressure [32,33,34,35].

The composition of the super-cytoplasm was quite sufficient for syntheses of amino-acids and of all other principal substances occurring in the cells of the present-day living matter, at least, in the cells of prokaryotes. We believe that the processes leading to the DNA and RNA syntheses could continue in the super-cytoplasm under the condition of the occurrence of CH_4_ and diffusion of nitrate and phosphate into the system. According to [57,63,64] (see also [86]), nucleic acids are capable of self-replicating. A possible mechanism of DNA self-replication is considered in [38,39,40]. One opinion, that the process of DNA self-replication should proceed by the autocatalytic mechanism, was proposed in [86]; it requires experimental confirmations. The confirmation of this assumption would mean that the ability of replicating is not the specific property that distinguishes nucleic acids from other polymers. The concentration of nucleic acids in the super-cytoplasm under appropriate conditions can increase in time. When it achieves some critical value, cells originate and the subsequent development of the replication processes within them and their division proceed, in general, similarly to the corresponding processes proceeding within the cells of the present-day simple organisms. D. Abel and J. Trevors note reasonably that convincing formulation of the mechanism of reproduction of hereditary characters is a very important requirement to any life origination hypothesis [82,83].

We suppose that the mechanism of formation of the cellular envelopes in the super-cytoplasm is as follows. It is well known that waters actively sorb around non-watered hydrogen bonds –H_2_N^+^····^−^O= [32,33,34,35]. Such bonds are characteristic for pair-wise joining of N-bases belonging to nucleosides, nucleotides, and nucleic acids in concentrated aqueous media. Although, in the super-cytoplasm, intermolecular hydrogen bonds have different degrees of watering, it may happen in any moment that several or many molecules actively sorbing waters are localized in a minor volume of super-cytoplasm. This event may lead to the occurrence of the shell zone of a decreased water concentration around these organic molecules. This water-concentration decrease can be sufficient for precipitation of an organo-phosphate semi-permeable shell at some distance from the molecules under consideration. If the water concentration over the system is rather low and the rate of formation of new organic molecules is rather high, a multitude of such shells may precipitate simultaneously. Later on, each cell can obtain water and organic substances only as a result of their diffusion through the cell membrane. Thus, the intracellular cytoplasm appears in addition to the super-cytoplasm. Now, the nucleic acids replicate inside the cells. They were the first proto-cells. Within any one localization, a multitude of similar but different proto-cells originate, containing similar but different DNA- and RNA-like molecules. The evolution process begins after origination of the proto-cells.

The basic features of the mechanisms (not of the chemisms) of the DNA replication, binary fission, and mitosis, within the terms of the LOH-hypothesis, are formulated by us fragmentarily in [1,39] and, in detail, in [38,40].

Apparently, there are grounds to believe that the simplest organisms just after their origination within the super-cytoplasm are characterized by rather weak functions of protein synthesizing and inheritance of principal characters. With time, these functions gradually develop and strengthen in the course of the evolution. The following facts count in favor of this assumption. First, DNAs of the simplest present-day organisms, such as the prokaryotes and viruses, contain (unlike DNAs of the eukaryotes) no protein and the cytoplasm of the prokaryotic cells, although they contain ribosomes, which are considered as workshops producing proteins on the basis of genetic instructions, have much poorer protein content than the cytoplasm of eukaryotic cells. Second, the prokaryotes and the simplest unicellular eukaryotes are very variable, their species are extremely different and they often mutate; viruses and viroids mutate even more frequently. Thus, the simplest organisms are characterized by very weak mechanisms of inheritance of principal characters and these characters vary often from one generation to another.

We stress once more that amino-acids could originate in the super-cytoplasm arising after melting of the hydrate structure and that the processes of origination of amino-acids require no additional source substances besides those occurring in the super-cytoplasm and in the gas phase over it. Below, we will show that this soup can contain some amount of ammonia along with other substances considered above.

The notion on self-replication of functional polymer molecules is one of the central points of the LOH-hypothesis. The opinions cited above [57,63,64,86], and the hypothetical mechanism considered in [38,40], count in favor of the DNA double-helix self-replication in nutrient media. However, the actual possibility of such a process is not evident and remains to be verified experimentally. At present, when the methods of syntheses of artificial DNA-like double helixes are available, the experimental testing of the assumption on the possibility of the DNA self-replication could be verified by experts in organic chemistry.

Thus, we assume that the sequence of the events that leads to living matter origination is as follows: (1) niter diffusion into hydrate structure; (2) formation of N-bases and riboses within structural cavities; (3) phosphate diffusion from outside into small structural cavities; (4) formation of DNA- and RNA-like molecules through polycondensation; (5) melting of a portion of the system and water-organic-phosphate super-cytoplasm formation; (6) formation of amino-acids and simplest organelles in the super-cytoplasm; (7) self-replication of nucleic acids and concentrating of the super-cytoplasm; (8) formation of proto-cells.

All processes of this cycle proceed at low temperatures, in any one CH_4_-hydrate locality, and as a result of diffusion of only two widely-distributed substances into the solid structure. The underground proceeding of these processes allows for fulfilling the conditions of the unity of the site of chemical transformations, constancy of the ambient conditions, and slowness and “thermodynamic front” for the entire process, and the occurrence of the hydrate structure provides the DNA configuration and monochirality.

## 3. Thermodynamic Aspects

The subsequent calculations are aimed at thermodynamic confirmation of the feasibility of formation of DNA-like molecules from methane, nitrates, and phosphates under natural conditions with use of no external energy and at thermodynamic consideration of different important particular problems that are related to the living matter origination in the context of the LOH-hypothesis. We present the results related to living matter origination on the basis of CH_4_ in more detail and present the results related to other hydrocarbons briefly to compare them with those related to methane.

First of all, let us show that CH_4_ and nitrates are chemically capable of producing the set of N-bases and riboses necessary for DNA and RNA formation. The fact is that this process consists of redox reactions. Therefore, not only the elemental composition of the source substances but also the valence state of the source elements is of prime importance for determining the feasibility of the process under consideration. When choosing the possible substances that could be the sources for living matter origination, we determined the minimum set of different minerals that are rather widely distributed in the Earth’s crust and are capable of forming the DNA and RNA molecules in any one localization. Methane (or another methane hydrocarbon) was chosen from considerations of equality between the sizes of the DNA and RNA functional groups and the cavities of the gas-hydrate structure, and nitrate-ion (in the composition of sodium or potash niter) was chosen because no other valence state of nitrogen but N^+5^ is capable of forming all necessary N-bases and riboses. The stoichiometric equations (3) and (4) for the chemical reactions of formation of the sets of N-bases and riboses that enter the DNA and RNA molecules, respectively, are presented below. These equations are written for hypothetical DNA and RNA fragments that contain a set of one Th, one Cy, one G, one Ad, and four DDRs and a set of one U, one Cy, one G, one Ad, and four DRs, respectively:

27 KNO_3_ + 39 CH_4_ = 27 KOH + 34 H_2_O + 6 N_2_ +

+ C_5_H_6_N_2_O_2_ (Th) + C_4_H_5_N_3_O (Cy) + C_5_H_5_N_5_O (G) + C_5_H_5_N_5_ (Ad) + 4 C_5_H_10_O_4_ (DDR) (3)

28.2 KNO_3_ + 38 CH_4_ = 28.2 KOH + 32.4 H_2_O + 6.6 N_2_ +

+ C_4_H_4_N_2_O_2_ (U) + C_4_H_5_N_3_O (Cy) + C_5_H_5_N_5_O (G) + C_5_H_5_N_5_ (Ad) + 4 C_5_H_10_O_5_ (DR) (4)

It is seen that DNA and RNA formation is accompanied with nitrogen and water evolution and that the total gas pressure decreases significantly in the course of these reactions.

The conditions of the LMSE origination within gas-hydrate structure differ significantly from the standard ones. Meanwhile, all calculations are performed for the standard conditions. The causes of this approximation are as follows. First, under the standard conditions, the changes in the partial molar Gibbs free energy in the processes of transition of guest particles into hydrate phases and back are small in magnitude as compared to those in the processes under consideration. Second, gas hydrates are substances of variable composition, their thermodynamic functions can be determined precisely for some ideal compositions only and the partial molar thermodynamic functions (especially, entropy) of the host and guests can vary for different non-stoichiometric compositions. Therefore, the available thermodynamic data provide no possibility for exact calculations and it is unlikely that such calculations will be possible in the near future. For almost all reactions considered in this paper, the molar changes in the standard Gibbs free energies are so great in magnitudes that, for the purposes of this paper, the standard values given below are quite sufficient.

The full list of the values of the standard molar enthalpy of formation Δ_f_H_j_^0^(T) and absolute entropy S_j_^0^(T) for all substances under consideration is given in [1,31,32]. The data on N-bases are taken from [10,11]. For DR, which belongs to RNA, only standard enthalpy of formation is available [87,88]; the complete set of unpublished thermodynamic data for DR was put at our disposal by Boerio-Goates [12] (the enthalpy value obtained by this author does not differ from the values published in [87,88]).

The first condition of living matter origination is the CH_4_-hydrate occurrence within the Earth’s crust. According to the hypothesis of the Solar System formation (PFO–CFO hypothesis) [18,19,20,21,22,23,24,25,26], the Earth’s crust in the Earth formation period contained only minor amounts of methane and other saturated hydrocarbons and the major portion of the Earth’s lower hydrocarbons was formed within the Earth’s crust from sorbed hydrogen and carbon dioxide liberated as a result of decomposition of carbonate minerals. The changes in the Gibbs free energy under standard conditions for the reactions of CH_4_, C_2_H_6_, and C_3_H_8_ formation are equal to −130.6, −192.1, and −263.6 kJ/mol, respectively. These decreases in the Gibbs free energy are sufficient for these reactions to proceed under the conditions of the Archaean Earth. Under the Earth’s crust, methane-hydrate deposits formed within the voids and within the localities of porous minerals on the basis of water formed in the reactions of hydrogen with carbon dioxide and water diffused from outside. In our days (by the end of 2008), the occurrence of about 1.3×10^11^ t of methane in the Earth’s crust (for 103 countries) is proven [89] and the major portion of this methane is in the form of methane-hydrate [90].

Let us generally consider the question on the feasibility of synthesizing the set of N-bases and riboses that is necessary for RNA formation on the basis of saturated hydrocarbons and niter with no external energy. Qualitative estimations of the feasibility of DDR formation will be given below.

It is well known that the molar ratios Ad/Th and G/Cy in DNA molecules and Ad/U and G/Cy in RNA molecules are equal to unity and each sort of DNA or RNA molecules is characterized by an individual molar ratio (Ad + Th)/(G + Cy) or (Ad + U)/(G + Cy), respectively. For bacteria, this ratio can be above or below unity; for higher organisms, the range of variations in this ratio is comparatively narrow, e.g., for most animals, it is usually between 1.3 and 1.5 (the Ad, G, Th, and Cy contents in the human sperm are 31, 19, 31, and 19%, respectively); and, for higher plants, it is between 1.1 and 1.7 [91].

The reaction of formation of the full set of N-bases and DR that is necessary for origination of one mole of RNA molecules can be written in the form

a_1_KNO_3_ + a_2_C_n_H_m_ = a_3_U + a_4_Ad + a_5_Cy + a_6_G + 4DR + a_1_KOH + a_7_H_2_O + a_8_N_2_ (5)

where C_n_H_m_ is the formula of a source aliphatic hydrocarbon and a_1_–a_8_ are the stoichiometric coefficients (the stoichiometric coefficients for KNO_3_ and KOH are the same). Equation (5) shows how many molecules of each of the source substances are consumed and how many molecules of each of the products are produced, counting on 4 DR molecules on average over the chain. This equation corresponds to the situation when oxygen of niter reacts completely; *i.e.*, O_2_ is not produced. Different species are characterized by different molar ratios (Ad + U)/(G + Cy). We can introduce the notation

r = (a_3_ + a_4_)/(a_5_ + a_6_) (6)

and express the stoichiometric coefficients a_3_–a_6_ of Equation (5) through r. We can also write

a_3_ = a_4_; a_5_ = a_6_ (7)

since, in RNA molecules, the molar ratio (Ad/U) = 1 and (G/Cy) = 1). In each RNA molecule, the number of N-base is equal to the number of D-ribose groups. Therefore,

2a_3_ + 2a_5_ = 4 (8)

From (6), (7), and (8), we obtain

a_3_ = a_4_ = 2r/(r+1); a_5_ = a_6_ = 2/(r + 1) (9)

After substitution of (9) into (5), we have

a_1_KNO_3_ + a_2_C_n_H_m_ = [2r(C4H4N2O2 (U) + C5H5N5 (Ad)) + 2C4H5N3O (Cy) + C5H5N5O (G))] /

/ (r + 1) + 4C_5_H_10_O_5_ (DR) + a_1_KOH + a_7_H_2_O + a_8_N_2_ (10)

Then, we fit the stoichiometric coefficients a_1_, a_2_, a_7_, and a_8_ for (10). These coefficients can be expressed as follows:

a_1_ = 1.6 + 7.6m/n − (3.6r + 4)/(r + 1) (11)

a_2_ = 38/n (12)

a_7_ = 15.2m/n − 20.8 − (7.2r + 8)/(r + 1) (13)

a_8_ = 0.8 + 3.8m/n − (10 + 8.8r)/(r + 1) (14)

Equation (9) with coefficients (11–14) was used to calculate the changes in the standard Gibbs free energy (∆_i_G^0^, where i is the reaction number) for the processes of formation of the full set of substances necessary for synthesis of RNA molecules from niter and different hydrocarbons. The calculations performed for the sets characterized by different r values allow the following conclusions. The changes in the Gibbs free energy for the reactions of niter with CH_4_, C_2_H_6_, C_3_H_8_, C_2_H_4_, and C_3_H_6_ are negative and rather great in magnitude and vary only slightly with the r value. For example, the ∆_i_G^0^ values for the reaction between niter and CH_4_ at r = 0.0625, 1.00, and 16.0 are equal to −8227, −8281, and −8336, respectively and the ∆_i_G^0^ values for the reaction between niter and C_2_H_6_ at r = 0.0625, 1.00, and 16.0 are equal to −6050, −6104, and −6159, respectively.

These results mean that the LMSEs could originate from methane hydrocarbons and niter at the expense of the internal energy of the source substances and that thermodynamics allows wide variations in relative yields of N-bases. Significant variations in the equilibrium relation between produced N-bases correspond to minute variations in the Gibbs free energy changes, *i.e.* several tens of kilojoules. The Gibbs free energy variations of such an order could result from variations in the reaction conditions and in the nature of reactants (e.g., from the replacement of KNO_3_ by NaNO_3_). This conclusion is important, because it shows that different sets of N-bases could originate in different historical periods in any one region or in any one historical period in different regions of the globe.

The ∆_i_G^0^ values for the reactions under consideration are so high in magnitude that there are no doubts that these reactions are thermodynamically feasible within the phases of hydrocarbon hydrates under real conditions. The greater the molecular mass of the saturated hydrocarbon, the smaller the magnitude of the decrease in the Gibbs free energy.

The previous calculation was performed for RNA formation, because the thermodynamic functions for DDR are not available. However, some qualitative estimates for the thermodynamic feasibility of DDR formation in the chemical system under consideration can be made. Let us consider the reaction of formation of Th and DDR from U and DR.

C_4_H_4_N_2_O_2_(cr) (U) + CH_4_(g) + C_5_H_10_O_5_(cr) (DR) =

= C_5_H_6_N_2_O_2_(cr) (Th) + C_5_H_10_O_4_(cr) (DDR) + H_2_O(lq) (15)

As the first approximation, we assume that the DDR entropy is equal to the DR entropy. Tabulated values give, approximately, Δ_15_S_j_^0^ = −84.21 J/mol K. The contribution of the entropy term to the Δ_15_G^0^ value, TΔ_15_S_j_^0^ = −25.11 kJ/mol. Now, we use the following approach. We suppose that Δ_15_G^0^ = 0 and calculate the Δ_f_H_j_^0^ value for DDR, −781.1 kJ/mol. This result, in combination with the tabulated data of the standard values, means that, for the reaction

C_5_H_10_O_5_(cr) (DR) + H_2_(g) = C_5_H_10_O_4_(cr) (DDR) + H_2_O(lq) (16)

∆_16_(Δ_f_H_j_^0^) = −16.09 kJ/mol. It is known that the reactions of such a type, in which hydrogen reduces organic substances, are characterized by negative enthalpy changes much higher in their magnitudes than the obtained value. This means that the magnitude of Δ_f_H_j_^0^ for DDR is apparently higher than 781.1 kJ/mol. Thus, it is highly probable that the products of oxidation of simple hydrocarbons by niters contain DDR together with Th, Ad, G, Cy, U, and DR. Relative contents of these components depend on the conditions.

Earlier, it was shown [32] that the interaction between hydrocarbons and niters with formation of only DR and nitrogen is also thermodynamically feasible. On the whole, the coupled reactions of Th, Ad, G, Cy, U, DR, and DDR formation from niter and CH_4_, C_2_H_6_, or C_3_H_8_ with liberation of N_2_ are feasible. The reactions of DR or DDR formation apart from Th, Ad, G, Cy, and U formation are also feasible. The reactions of Th or U formation from niter and each of these hydrocarbons with liberation of N_2_ can proceed by themselves; however, Ad, G, or Cy cannot form by itself by such a mechanism.

We also studied the thermodynamic feasibility of reactions between simple hydrocarbons and niter proceeding with O_2_ liberation [32]. Calculations led us to the following conclusions. Under thermodynamic equilibrium, the complete set of N-bases or any one of N-bases, other than Th and U, can coexist with oxygen; however, the occurrence of gas oxygen excludes the occurrence of riboses. The complete set of N-bases and DR (and DDR) is not compatible with the occurrence of gas O_2_. The niter deficiency promotes formation of the full set of substances necessary for DNA (or RNA) formation. Such reactions proceed with N_2_ liberation. In reality, the chemical filling of a hydrate structure is determined by the NO_3_^−^ concentration (more precisely, by the NO_3_^−^ activity) in the environment and by the exposure time.

Consider some specific features of the reactions under consideration. Let the hydrocarbon pressure over hydrates be determined by the temperature independently of the fractional conversion of the hydrocarbon; the super-stoichiometric H_2_O converts to liquid phase. The gas pressure over a gas-producing reaction zone located under a rather penetrable stratum or under a seabed is self-regulating because the stratum acts as the pressure valve throwing off the pressed gas into the atmosphere or seawater. The point is that the LMSE are the products of incomplete interaction between hydrocarbon and niter within the hydrocarbon-hydrate phase. In reality, the above-considered reactions between CH_4_ and NO_3_^−^-ions are not necessarily equilibrium. The fact is that the complete nitrogen reduction could lead to ammonia formation:

KNO_3_ + CH_4_ = CO_2_ + NH_3_ + KOH + 295.7 kJ/mol (17)

The reactions proceeding according to (10) liberate N_2_; the gas pressure over the hydrate phase increases up to a limit level, which is determined by the hydrate-bedding depth, temperature of the hydrate phase, and density of the over-hydrate soil layer and seawater column. Then, the N_2_–CH_4_ mixture is thrown off into the atmosphere or seawater. The CH_4_ pressure remains at the initial level, and the throwing off of the N_2_ that did not achieve the highest degree of reduction to NH_3_ inhibits complete oxidation of carbon and thus stimulates stabilization of the produced LMSEs.

In a closed system, *i.e.*, under conditions when N_2_ is not thrown off from the reaction zone in the course of the reaction between niter and a hydrocarbon, this reaction goes through the state of the system that can be described as the equilibrium between initial substances (niter and hydrocarbon) and such products as N-bases, riboses, and NH_3_. For example, when the hydrocarbon is methane, we can write

23.25 KNO_3_ + 38 CH_4_ = C_4_H_5_N_3_O + C_5_H_5_N_5_O + C_5_H_5_N_5_ + C_4_H_4_N_2_O_2_ + 4 C_5_H_10_O_5_ +

+ 23.25 KOH + 22.5 H_2_O + 8.25 NH_3_ (18)

For this reaction, Δ_18_G^0^ = −6146 kJ/mol. We see that the full set of the LMSE can be formed from CH_4_ and KNO_3_ with liberation of NH_3_. For reactions of such a type with C_2_H_6_ and C_3_H_8_, the Δ_i_G^0^ values are also negative, −5198 and −4757 kJ/mol, respectively.

In natural situations, most of the equilibria considered above cannot be reached. The natural reactions of a hydrocarbon with a niter can proceed in a complicated way with liberation of N_2_, O_2_, NH_3_, and CO_2_. Decreasing in the Gibbs free energy shows that a reaction proceeds in the direction determined by the written stoichiometric equation; however, it does not show that the substances written in the left-hand side of the equation react only by the direction that corresponds to this equation.

Consider one more question of the DNA structure that can be answered on the basis of a thermodynamic consideration. Why do only five N-bases usually enter the DNA and RNA compositions, and why are the other ones random? To understand the principle that could underlie this “lusus naturae” (caprice of Nature), we consider the reaction between guanine and water with formation of xanthine. Let the reactions leading to formation of N-bases and riboses proceed in a closed system and an equilibrium be established after a time. It is clear that equilibrium in the reaction system suggests equilibrium between all its components, in agreement with the detailed equilibrium principle; this principle allows us to elucidate whether X can exist in the system containing G and H_2_O. Consider the reaction

C_5_H_5_N_5_O (G) + H_2_O = C_5_H_4_N_4_O_2_ (X) + NH_3_ (19)

proceeding under standard conditions. Calculations give ∆_19_G^0^ = 7.32 kJ/mol. This estimate means that equilibrium (19) is shifted to the left and X formation is thermodynamically disadvantageous. Apparently, the analogous cause hampers entry of other N-bases other than Ad, G, Th, Cy, and U into DNA and RNA molecules. The absolute change in the free energy is small; therefore, nucleic acids may contain X under certain conditions differing from standard ones. Indeed, X sometimes enters the compositions of natural nucleic acids. This consideration confirms the above-formulated conclusion that the gas mixtures formed in the process of LMSE origination could contain NH_3_.

The thermodynamic consideration given above allows the following important conclusions: (i) only +5-valence nitrogen in combination with −4-valence carbon can provide formation of the entire set of N-bases and riboses necessary for DNA and RNA origination; (ii) reactions between niter and CH_4_, C_2_H_6_, or C_3_H_8_ at temperatures in the vicinity of 298 K can lead to formation of all N-bases inherent in DNA and RNA molecules, DR, and DDR; (iii) the relative yields of these substances depend on the conditions and can vary in wide ranges; (iv) just the thermodynamics is instrumental in the selection of N-bases to be further incorporated in nucleic acids; (v) formation of N-bases, DR, and DDR from hydrocarbons and niters is associated with liberation of O_2_, or N_2_, or NH_3_ or with simultaneous liberation of O_2_, N_2_, NH_3_, and CO_2_ and with enrichment of the Archaean atmosphere with these gases in proportions dependent on the underground reaction conditions; (vi) a portion of NH_3_ that liberates in the process of formation of N-bases can be used for formation of amino-acids; and (vi) the reactions between niters and hydrocarbons can proceed in different reaction zones over the globe and in different time periods.

The fulfillment of the following main conditions is necessary for LMSE origination on any planet. First, the crust of the planet should contain water, compact deposits of hydrogen or substances capable of decomposing with H_2_ liberation, carbon dioxide or carbonates capable of decomposing with CO_2_ liberation, niters or other sources of +5-valence nitrogen, and phosphates or another source of phosphorous capable of oxidizing to phosphate. Second, the temperature within the planet's crust should be appropriate for formation and long-term stability of hydrocarbon-hydrates, niters or other substances containing +5-valence nitrogen, and phosphates and should provide the running of chemical reactions between minerals and organic substances.

## 4. Discussion

The LOH-hypothesis differs principally from all living matter origination hypotheses published earlier. These differences are not only factual but also represent a different world-outlook and philosophy. We consider the life origination process as a sequence of thermodynamically caused regular and inevitable chemical transformations, which are regulated by universal physical and chemical laws. We assume that living matter originated repeatedly in different localizations, and that each localization could give rise to a multitude of different living organisms. Different organisms consist of cells that are similar in their constitution, because they are built by Nature on the basis of the same mineral materials and the same physical and chemical laws.

Not proteins nor amino-acids, but DNA and RNA molecules are the first carriers of life. Living matter originated and can originate now everywhere where necessary minerals and necessary ambient conditions exist and where these conditions are continuous for long periods of time.

Our hypothesis includes an important notion of a “thermodynamic front” whose temporal movement determines the slow (on the human life duration scale) stepwise filling of the gas-hydrate cavities, *i.e.*, formation of purine and pyrimidine nuclei within the large cavities, of riboses within small cavities, and of substituting groups in the purine and pyrimidine nuclei and subsequent formation and lengthening of the DNA- and RNA-like molecules.

It is an open secret that Darwin’s evolutional hypothesis on the stick-slip development of living matter from the simplest organisms to highly organized ones limps and the limping does not become less evident with time. The notion of evolutionary accumulation of minor changes in the DNA of an organism and revolutionary manifestation of summed minor changes in appearance of a new species is scarcely justified.

The experiments with drosophila and other rapidly reproducing species have beenperformed widely around the world for more than half a century. Let us take that 50 researchers perform such experiments for 50 years. This means that 50×50×18 = 4.5×10^4^ generations were studied. It is more likely that the last value is underestimated. This result is approximately equivalent to the case when any one allele is studied for 4.5×10^4^ generations; for such a period, no new stable species has been observed in spite of wide variations in experimental conditions. For a higher organism, for which the generation period can be 10 years or more, this number of generations responds to about 5×10^5^ years. Each researcher is capable of observing more than one fly family simultaneously. Therefore, these numerals should be multiplied by a factor of several units. Taking into account that living matter exists on Earth for no more than (3.5–4.0)×10^9^ years, it is logical to doubt Darwin’s opinion that Nature created simplest living organisms, that their heritage varied from generation to generation as a result of mutations and natural selection, and that the generations with novel stable characters could originate new superior and more complicated species till the appearance of mammals, including *Homo sapiens*.

According to Darwin, the fossil record should be rife with examples of transitional forms leading from the less to more evolved species. Quite the contrary, “…instead of filling the gaps in the fossil record with so-called missing links, most paleontologists found themselves facing a situation in which there were only gaps in the fossil record, with no evidence of transformational intermediates between documented fossil species” [92]. The Darwinian hypothetical evolutionary chains have a number of wide gaps, such as between primitive terrestrial mammals and whales or between molluscs and arthropods. However, as was pointed in [93], the punctuationist hypothesis on the possibility of rapid interspecific evolutionary transformations resulted from random mutations has neither logical nor geological grounds. It was mentioned above that, according to [72], multicellular organisms appeared independently in the Earth history no less than 24 times. Meanwhile, the today life-origination hypotheses, at least those actively discussed in the scientific literature, are not able to explain the plurality of the life origination phenomenon, because they consider living-matter origination as a random, rare, and single event and, furthermore, most of them proceed from the incorrect principle of the necessity of external energy for synthesis of biologically-active substances from minerals.

Principally a new explanation of the variety of living-matter species becomes more necessary over the years. The LOH-hypothesis allows for explaining the living-world variety from naturalistic positions. As was said above, we assume that the DNA and RNA molecules originated from minerals within the natural underground incubators, where they had been forming and upgrading with time and had only a minor contact with the outside. Little by little, they increased in length and their thermodynamic ordering improved. Thus, the longer the period of DNA and RNA formation within the hydrate matrix, and the later the hydrate matrix melted and the DNA and RNA molecules got to ambient water–mineral or soil–atmosphere media, the more complicated organisms developed on the basis of the DNA and RNA molecules matured in their incubators. In other words, we can say that different species of living organisms originated in the period of formation of the DNA and RNA molecules. Perfecting of the primary DNA and RNA molecules in the underground incubators could be interrupted occasionally, and, thus, the complication of the species resulted from further transformations of the nucleic acids was not continuous. Therefore, filling the gaps in the Darwinian series is, seemingly, unpromising. It is quite possible that Darwin’s hypothesis was of significance for the survival and adaptation of organisms to the conditions of ambient media, but it scarcely was the leading one for principal transformations of species.

It is known that the simpler the organisms, the stronger their variability with time. Apparently, the cause of enhanced variability of simple organisms lies in the fact that the DNA molecules that govern their heritage were housed insufficiently long in natural incubators and, therefore, they were insufficiently ordered in the thermodynamic understanding of this term.

The LOH hypothesis gives grounds for an entirely new explanation for the chiral purity of DNA, RNA, and their derivatives. We suggest that the chiral purity of nucleic acids is a consequence of the effect of the geometry of the matrix in which they were first molded in nature and have continued to reproduce themselves in each newly formed cell of living matter. Different enantiomers of ribose have different geometries responsible for the chiral purity of DNA and RNA. Apparently, only D-riboses can “touch” both an N-base and a phosphate group, by the active groups of their structures. Only under such conditions, is each phosphate group capable of “touching” two riboses and thus of joining together two nucleotides. It may be expected that this intriguing issue will be resolved in computer experiments. As for L-riboses, they allow formation of amino-acids and other side products.

Maybe, there are grounds to consider the possibility of living-matter origination within honeycomb structures of phosphates. However, this task is beyond the theme of this paper, because we suppose that this mechanism is of low probability for several reasons.

It would seem that the possibility of living-matter formation within the zeolite structures should also be considered. However, had such clumsy molecules as nucleic acids actually formed within zeolites, they would have found it very difficult to make their way out. Things are made still easier for nature by the fact that the gas-hydrate matrix, in which DNA and RNA molecules are supposed to have formed, contained initially two sorts of atoms (carbon and hydrogen) of the five ones necessary for DNA and RNA synthesis.

## 5. Conclusions

Thus, according to the LOH-hypothesis, living matter originated as a result of the following evolution sequence.

**Figure 3:**
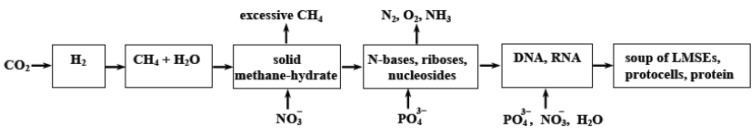
The living-matter origination evolution sequence according to the LOH-hypothesis.

The LOH-hypothesis is the novel naturalistic hypothesis of living matter origination, which has been developed by us for about ten years; it has no common features with any available hypothesis that considers this problem. In particular, the LOH-hypothesis evolution sequence differs principally from the evolution sequence (1) proposed by Oparin.

In our opinion, this hypothesis is applicable for any planet of the Universe where the conditions for realization of the proposed mechanism of living matter origination exist.

A number of the LOH-hypothesis positions have approximate thermodynamic substantiations.

The LOH hypothesis allows an unexpectedly simple and experimentally verifiable assumption about the nature of monochirality of DNA and RNA molecules.

The hypothesis under consideration corresponds to six common principles formulated in the Introduction to this paper.

In our opinion, the long-term availability of the following three factors in an underground or under-seabed region is necessary and sufficient for origination of nucleic acids and proto-cells: (1) the methane-hydrate mineral matrix (possibly, the hydrate of any other hydrocarbon is also suitable); (2) two additional mineral substances (niter, and phosphate) that are capable of selective chemical interaction with methane; and (3) the temperature level of the environment (273 ± 20 K) so low that the chemical reactions in the system could represent the step-by-step sequence controlled by the “thermodynamic front”.

The LOH-hypothesis contains a number of hypothetical issues, which could become the subjects of further experimental and computer studies. The first of them is the possibility of self-replication of the double DNA-like molecules within the organo-phosphate aqueous solutions. A group of tasks can be put for computer simulation of rearrangement of different LMSEs and DNA- and RNA-like molecules within the hydrate structures II and H; it should be taken into account that the tabulated parameters of the hydrate structure can somewhat vary depending on the ambient conditions and the guest nature. Some of the tasks of such a kind are mentioned above. A possible laboratory experiment on autoclave syntheses of LMSEs is described in [1,31]. In our opinion, this important, rather expensive, and not simple experiment should be performed on an international basis.

Several available observational and experimental data count in favor of the LOH-hypothesis. Some interesting observations are described in [94,95,96,97] and generalized in [31]. Note also the experimental work [98], where it was stated that the synthesis of pyrimidines from methane and urea does not go in liquid water, but goes in the water ice phase, although, it would seem that cooling should decrease the rates of chemical reactions. It is quite possible that the observed reactions were stimulated by the formation of gas-hydrate structure within the icy reaction medium.

The experimental studies performed in the last decades show that the chemical nature of the polymer-forming group in different genomes is variable. Namely, different genomes may contain, along with the more abundant PO_4_^3−^ group, the AsO_4_^3−^ [99] and PO_3_S^3−^ [100] groups, which are close to the PO_4_^3−^ group in their sizes, chemical activities, and thermodynamic characteristics. In our opinion, this shows that chemical composition of this group is not of crucial importance for living-matter formation; this group is only a “bridge” that joins nucleosides in the DNA and RNA sequences. However, the sizes of this bridge group should not exceed the sizes of the gas-hydrate small cavities. The chemical composition of this group depends on the chemical composition of the environment.

Finishing the paper, we note one possibility that might seem to some people to be just an idle fancy. The point is that, apparently, a combination of methane, niter, phosphate, and water represents the simplest set of nutrients to maintain life for some organisms at sufficiently low temperatures. By varying the temperature and nutrient composition, the rates of the metabolic processes can be decreased many-fold and regulated; therewith, the waste can be minimized. It cannot be excluded that the cellular life could be prolonged due to deceleration of cellular division and that, after a time, the usual rate of the cellular metabolism can be restored by returning to the usual nutrient and conditions. In our opinion, this is an interesting theme for studies, which could be used for verification of our hypothesis.
